# Identification of Growth-Associated Genes by Genome-Wide Association Study and Their Potential Application in the Breeding of Pacific White Shrimp (*Litopenaeus vannamei*)

**DOI:** 10.3389/fgene.2021.611570

**Published:** 2021-04-07

**Authors:** Ding Lyu, Yang Yu, Quanchao Wang, Zheng Luo, Qian Zhang, Xiaojun Zhang, Jianhai Xiang, Fuhua Li

**Affiliations:** ^1^Key Laboratory of Experimental Marine Biology, Institute of Oceanology, Chinese Academy of Sciences, Qingdao, China; ^2^Laboratory for Marine Biology and Biotechnology, Qingdao National Laboratory for Marine Science and Technology, Qingdao, China; ^3^Center for Ocean Mega-Science, Chinese Academy of Sciences, Qingdao, China; ^4^Yantai Institute of Coastal Zone Research, Chinese Academy of Sciences, Yantai, China; ^5^University of Chinese Academy of Sciences, Beijing, China; ^6^The Innovation of Seed Design, Chinese Academy of Sciences, Wuhan, China

**Keywords:** GWAS, growth trait, marker assisted BLUP, genetic breeding, *Litopenaeus vannamei*

## Abstract

The Pacific white shrimp (*Litopenaeus vannamei*) is the most widely cultured shrimp in the world. A great attention has been paid to improve its body weight (BW) at harvest through genetic selection for decades. Genome-wide association study (GWAS) is a tool to dissect the genetic basis of the traits. In this study, a GWAS approach was conducted to find genes related to BW through genotyping 94,113 single nucleotide polymorphisms (SNPs) in 200 individuals from a breeding population. Four BW-related SNPs located in LG19 and LG39 were identified. Through further candidate gene association analysis, the SNPs in two candidate genes, *deoxycytidylate deaminase* and *non-receptor protein tyrosine kinase*, were found to be related with the body weight of the shrimp. Marker-assisted best linear unbiased prediction (MA-BLUP) based on the SNPs in these two genes was used to estimate the breeding values, and the result showed that the highest prediction accuracy of MA-BLUP was increased by 9.4% than traditional BLUP. These results will provide useful information for the marker-assisted breeding in *L. vannamei.*

## Introduction

The Pacific white shrimp, *Litopenaeus vannamei*, is the most widely cultured shrimp all over the world (FAO, 2019). Due to its extremely high industry value, genetic improvement by selection breeding has been performed for decades (Argue et al., 2002; Sui et al., 2016). Growth trait is considered as the most important economic trait in *L. vannamei* and has been taken as the main target trait in most breeding programs (Argue et al., 2002; Sui et al., 2016).

As a typical quantitative trait with medium to high heritability (Castillo-Juárez et al., 2007; Sui et al., 2016; Nolasco-Alzaga et al., 2018), improvement of the growth trait was performed mainly by best linear unbiased prediction (BLUP) (Castillo-Juárez et al., 2007; Sui et al., 2016; Nolasco-Alzaga et al., 2018), which is essentially based on an “infinitesimal model” (Mather, 1941). This model assumes that the quantitative trait is controlled by an infinite number of gene loci that are evenly distributed across the genome and each locus has an infinitely small effect (Fisher, 1918; Bulmer, 1971). However, substantial advances in molecular genetics and genomic research provided us an opportunity to capture the quantitative trait loci (QTLs) or genes that have definite effects on growth.

An essential method for identifying QTLs is genetic linkage mapping. Several studies based on this method have identified QTLs for growth trait in *L. vannamei*, and no related genes were found due to the limitation of marker density (Andriantahina et al., 2013; Yu et al., 2015). Besides, QTLs detected in a mapping population with a biparental family are usually difficult to be transferred to large, more complex breeding groups (Takeda and Matsuoka, 2008). With the development of high-throughput genotyping platforms for single nucleotide polymorphisms (SNPs), genome-wide association study (GWAS) has become a more powerful tool to dissect the genetic basis underlying the traits. It utilizes high-density SNPs distributed along the genome to determine the QTLs by statistical associations between SNP genotypes and particular phenotypes. GWAS has been successfully carried out in fish, referring to many traits, such as filet texture, fat content (Sodeland et al., 2013), growth (Gonzalez-Pena et al., 2016; Li et al., 2017), sexual maturation (Gutierrez et al., 2015), disease resistance (Liu et al., 2015; Zhou et al., 2017; Rochus et al., 2018; Tan et al., 2018), low oxygen tolerance (Zhong et al., 2017), and fatty acid composition (Horn et al., 2020). As for crustaceans, reports to use GWAS to discover SNPs or genes related to interesting traits are relatively less. Based on the restriction-site-associated DNA (RAD) sequencing data in a biparental family, GWAS analysis for growth trait was performed and two growth-related candidate genes *protein kinase C delta type* and *ras-related protein Rap-2a* were identified in *L. vannamei* (Yu et al., 2019). In another GWAS analysis, 23,049 SNPs were obtained in a population of 200 *L. vannamei* using 2b-RAD sequencing, and *class C scavenger receptor* was identified as a growth-related candidate gene (Wang et al., 2019). Then, as the genome was assembled, another growth-related candidate gene, *MMD2*, was found based on the correlation analysis results in Wang et al. (2019) and further validated (Wang et al., 2020). Although these researches illustrated the power of identifying QTLs or genes using GWAS in *L. vannamei*, low marker density and simple population structure restrict their applied values.

With the accomplishment of the whole genome sequencing in *L. vannamei* (Zhang et al., 2019), performing GWAS analysis and screening trait-related genes with reference genome and high-density SNPs become possible. In the present study, the 2b-RAD sequencing data were mapped to the published genome sequence, and the SNPs were called based on the reference sequence, then GWAS for body weight (BW) was performed. Candidate growth-associated genes were further validated in other populations. Moreover, a trial to apply marker-assisted selection (MAS) was conducted. These results will provide useful information for marker-assisted selection in *L. vannamei*.

## Materials and Methods

### Identification of Growth-Associated Markers by GWAS

The experimental population used in GWAS was the same as described previously (Wang et al., 2017, 2019). Briefly, the GWAS population which was named as population 1, consisting of 13 full-sib families, was bred in Guangtai Marine Breeding Company in Hainan Province of China in 2015. Each family was raised separately for seedlings. After grown to 3 cm, 50 individuals per family were randomly selected, mixed, and cultured in a 10-m^2^ pond. When they reached the market size, 200 shrimp were selected randomly to be taken as GWAS population and the BW of each shrimp was measured. The 2b-RAD library sequencing data of these 200 individuals have been published previously (Wang et al., 2017, 2019), and 23,049 SNPs were obtained in these researches. As the reference genome has been finished and published recently (Zhang et al., 2019), the sequencing data were mapped to the genome sequence, and the SNPs were called based on the reference sequence. After quality control filtering by minor allele frequency at 0.05 and SNP call rate at 0.9, 94,113 SNPs were used in subsequent GWAS.

Considering a possible stratification due to multiple origins of the shrimp, GWAS was performed using the egscore function in GenABEL package implemented in R environment (Aulchenko et al., 2007). In this method, classic principal component analysis (PCA) of the genomic kinship matrix was used to evaluate the population stratification (Price et al., 2006). Then, genome-wide significance was assessed by the Bonferroni method, in which the conventional *p* value was divided by the number of tests performed (SNPs tested). The model used for GWAS was as follows:

y=Xb+Ws+e,

where *y* is the vector of phenotypic records (BW), *b* is the vector of fixed effects including sex and the first four principal components, *X* is the incidence matrices for fixed effects in *b*, *s* is the effect of the SNP genotype, *W* is the design vector for *s*, and *e* is the vector of random residuals.

### Validation of Growth-Associated SNPs by the General Linear Model

After GWAS, another independent population bred in 2017 which was named as population 2 in this study was used to validate the identified growth-associated SNPs. Population 2 contains 303 individuals from multiple families with similar day age. The top 50 candidate SNPs with the smallest *p* values obtained in GWAS were genotyped in population 2 by the target sequencing method (Shanghai Biowing Applied Biotechnology Co., Ltd., Shanghai, China). The general linear model (GLM) was used to verify associations between them and BW in population 2. The GLM was as follows:

$$y=X_{1}b_{1}+Ws+e,$$

where *y* is the vector of phenotypic records (BW), *b*_1_ is the vector of sex fixed effects, *X*_1_ is incidence matrix for sex fixed effect in *b*, *s* is the effect of the SNP genotype, *W* is the design vector for *s*, and *e* is the vector of random residuals. The GLM analyses were executed using the package *stats* in R.

### Annotation of Significant SNPs

After validation, the upstream 10 kb and downstream 10 kb around the significant SNPs were blasted to *L. vannamei* genome^1^ and the nearby genes were identified. The 5′ untranslated regions (UTRs) and open reading frames (ORFs) of growth-associated candidate genes were predicted by the Expert Protein Analysis System (EXPASY^2^).

### Identification of Significant SNPs in Candidate Genes by the Mixed Linear Model

To identify SNPs associated with BW located in two candidate genes, the population bred in 2018 which was named as population 3 was used to genotyped SNPs located in 5′ UTR of the candidate genes. Population 3 contained 321 individuals from more than 50 full-sib families with the same day age. Mixed linear model (MLM) analysis was applied to eliminate the effects of population structure. Twelve microsatellite markers (Table 1) were used to genotype this population and their mothers to reconstruct pedigree in the COLONY (Jones and Wang, 2010). The restricted maximum-likelihood method was carried out in ASReml (Gilmour et al., 2012) to solve the following MLM:

$$y=X_{1}b_{1}+Zu+Ws+e,$$

**TABLE 1:** Primer sequence used for genotyping the microsatellites.

Microsatellites	Forward primer sequence (5′–3′)	Reverse primer sequence (5′–3′)
M1590	TCCCTTTGCATCTTGCTCTT	GAAGGTGAGGGAAGGGGTAG
M13810	GACGGTGACTTGAAGGGTGT	TGCACCGTCTTTGTAATCCA
M6687	GAGTTGAAGAAGGAAATGTCGG	TTTCTCTTTGCCCATTGTCC
M5476	GGCGAAGAAATAGCAACCAA	TTCGACACGCCTAGTACACG
M24175	CAGCTTCCATTTCACGAACA	CAAAGAAGATGAAGACATAGGCAA
M12607	AGCGTAGCACCTCTGCATTT	AAGAGGAAGACGAAGGAGGC
M21670	TGCTATCATTACTACCACTGATACTGC	TCTCTCGACCCAGTGAAAGG
M2882	CGACAGCTGTTGCTCAAGAC	AAAAGGTCCTTCGGGAGTGT
M11263	CTTAATCTTGGAAGCCAGCG	CGAAAAACAAGAAGATACAAACGA
M15481	CCATTCTCATTCTCATTCTCTTTTT	AAGATGGCAATTCCAACAGC
M13347	CCATTCTCAAGCCCATTGTT	GTTGATGGTGGTGGTTGATG
M9990	CACGAAGAAGAAGACGAAGGA	AGCATAGGCGCTAGAACAGC

where *y* is the vector of phenotypic records (BW); *b*_1_ is the vector of sex fixed effects; *X*_1_ is the design matrices for *b*; *u* is the random polygenic additive genetic effect except for target SNP, assumed to be $∼N\left(0,Z\sigma_{u}^{2}\right)$, where $\sigma_{u}^{2}$ is the polygenic additive variance; *Z* is the additive genetic relationship matrix for *u*; *s* is the fixed effect of the SNP genotype; *W* is the design vector for *s*; and *e* is the vector of random residuals.

After MLM analysis, linkage disequilibrium (LD) analyses were performed for BW-associated SNPs using the package LDheatmap in R, and additive (*a*), dominance (*d*), and substitution (*α*) effects of each BW-associated SNP were calculated as follows (Falconer and Mackay, 1996):

a=(AA-BB)/2

d=AB-(AA+BB)/2

α=a+d(p-q)

where *AA*, *BB*, and *AB* were the least squares means of BW of three genotypes, and *p* and *q* are the allele frequencies of *A* and *B*.

### Comparison Between BLUP and Marker-Assisted BLUP

After obtaining SNPs located in candidate growth genes, the methods of MAS were further explored. We calculated the estimated breeding values (EBVs) based on marker-assisted BLUP (MA-BLUP). The EBV of an individual based on MA-BLUP is the sum of polygenic effect ($\hat{u}$) and SNP genotype effects ($\hat{s}$.) which were obtained by solving MLM equations:

$$EBV=\hat{u}+\hat{s}.$$

For comparison, EBVs from traditional BLUP model were also obtained using a similar MLM but without SNP effects. Tenfold cross-validation was used to compare the accuracies of BLUP and MA-BLUP. The GLMs were also included in the comparison, which represented selection accuracies based on significant markers. The phenotypes under GLMs were predicted by fixed effects of sex and SNP genotype, which were the same as those used in the validation of growth-associated SNPs. Cross-validation was performed in population 3. For each tenfold cross-validation, the dataset was randomly divided into 10 equal zed subsets. Nine subsets were used as training data and the other one is used as prediction data. Cross-validation was repeated 500 times. Accuracy was represented by average Pearson correlation between observed phenotypes and predicted phenotypes in the prediction dataset.

## Results

### Genome-Wide Association Analysis

**FIGURE 1:**
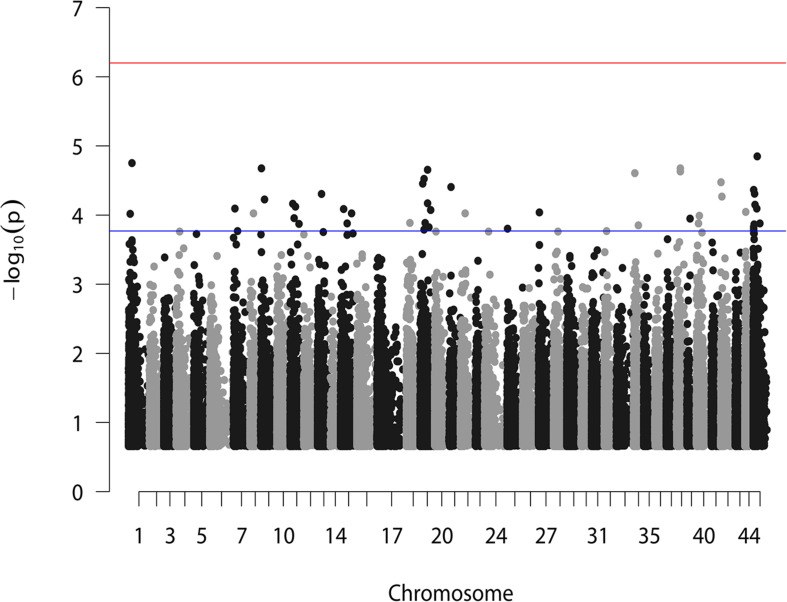
GWAS analysis for BW of *L. vannamei*. Two horizontal lines represent the genome-wide significant threshold (6.27) and the top 50 most significant threshold (3.77).

According to the reference genome information (Zhang et al., 2019), a total of 83,015 SNPs were distributed along the 44 linkage groups (LGs) with one SNP per 0.05 cM on average, while the other 11,098 SNPs were not assigned to any LGs. The Manhattan plot of the GWAS result is shown in Figure 1, in which the unassigned SNPs are shown in the rightmost column. We selected the 50 SNPs with the smallest *p* values for further verification. Their *p* value ranged from 1.42E–05 to 1.71E–04. A total of eight significant SNPs were located in LG19, and four and three were located in LG11 and LG15, respectively. The other 24 SNPs were located in 17 different LGs. Therefore, potential trait-associated SNPs showed a wide distribution in genome. More details about these 50 SNPs are shown in Table 2.

**TABLE 2:** Top 50 most significant SNPs and their alleles and *p* values.

ID of SNPs	LG	Alleles	*p* value	ID of SNPs	LG	Alleles	*p* value
SNP3484_497845	*	A > G	1.42E–05	SNP2218_1256681	19	C > T	8.41E–05
SNP3259_421418	1	G > A	1.78E–05	SNP2646_123180	44	G > A	8.96E–05
SNP3079_462600	9	A > G	2.11E–05	SNP2622_112991	27	T > C	9.09E–05
SNP629_59929	38	T > A	2.11E–05	SNP2637_447054	8	T > A	9.41E–05
SNP1678_890804	19	G > A	2.20E–05	SNP3361_1326159	15	C > A	9.42E–05
SNP3079_462612	38	T > C	2.34E–05	SNP3019_582769	22	G > A	9.44E–05
SNP1479_138609	34	G > C	2.48E–05	SNP696_176477	1	T > C	9.60E–05
SNP3406_465799	19	A > G	3.00E–05	SNP2022_375467	40	T > A	1.02E–04
SNP3413_17145	42	T > C	3.33E–05	SNP813_483296	11	T > C	1.10E–04
SNP994_314341	19	G > A	3.50E–05	SNP2570_399791	39	G > C	1.13E–04
SNP555_246199	21	C > G	3.94E–05	SNP2550_595246	19	T > A	1.30E–04
SNP555_246201	21	G > C	3.94E–05	SNP3319_422578	18	T > G	1.31E–04
SNP905_41442	*	G > C	4.33E–05	SNP776_802476	15	C > T	1.32E–04
SNP3981_169565	*	A > T	4.92E–05	SNP3635_782287	*	C > A	1.32E–04
SNP1809_497621	13	C > T	4.95E–05	SNP3424_278362	40	G > A	1.32E–04
SNP2152_130229	42	A > T	5.38E–05	SNP2698_1065913	11	T > G	1.34E–04
SNP1824_464525	9	G > A	5.91E–05	SNP2056_91598	*	G > A	1.36E–04
SNP1678_860109	19	G > A	6.79E–05	SNP2032_580787	34	A > T	1.41E–04
SNP1257_359745	11	T > C	6.87E–05	SNP811_40727	*	A > G	1.46E–04
SNP3085_190487	*	T > C	7.06E–05	SNP1678_1042666	19	C > T	1.50E–04
SNP813_657421	11	G > C	7.52E–05	SNP3136_56353	25	G > A	1.57E–04
SNP2315_402243	*	A > G	7.99E–05	SNP4197_33580	*	A > T	1.58E–04
SNP2315_402245	*	T > C	7.99E–05	SNP3406_444940	19	C > T	1.62E–04
SNP2930_199692	7	A > G	8.10E–05	SNP4619_34295	*	T > A	1.64E–04
SNP2591_392420	15	G > A	8.13E–05	SNP1233_275219	32	C > T	1.69E–04

The two numbers separated by underscore in SNP ID represent the ID of the scaffold and position of SNP in this scaffold (bp). For sequences of scaffolds, see http://www.shrimpbase.net/vannamei.html. The asterisk (*) indicates LGs of these SNPs were unknown.

### Validation of Growth-Associated SNPs by GLM

Eight candidate SNPs with small *p* values in GWAS were amplified and genotyped in population 2 by using the target sequencing approach. Their primer sequences are shown in Table 3. As there is a high ratio of repeat sequences and insertion–deletion mutations, the other 42 candidate SNPs were not genotyped. The GLM analysis results are listed in Table 4. Minor allele frequencies in all except for SNP555_246199 were larger than 0.10. The results of GLM analyses indicated that four of them were significantly correlated with BW after Bonferroni correction.

**TABLE 3:** Primer sequence used for genotyping candidate SNPs in validation population.

ID of SNPs	Forward primer sequence (5′–3′)	Reverse primer sequence (5′–3′)
SNP3085_190487	TCGCACCGGAAGATTATAGATATG	AAAGCGAATATAAAAGAGGGAAGG
SNP1678_860109	CTCCACCCTTGCCCATCTC	ACTCAGACACTTCTGGCGTAATAA
SNP3406_465799	TCGAAGGCAGGATATAATCATAGG	AACTCTCCTTGCCAAGTCAG
SNP2570_399791	AAAAACATCATAACGGTCACTCG	AAAATGTTTGTTGCGTGAGGATG
SNP3424_278362	AAGAGTAGAGAAGCAGAAGAGAAG	ATAATGCCATACCGCTTTGATATC
SNP555_246199	TCTTAACAATGCTAAATGGGGAG	TACCAACAAATCGCAAAATAAATC
SNP629_59929	TGAGTCTCCTGCTTTAAGGTGC	CAATGGTACTTTATTTAAGTCACTGAG
SNP2315_402243	ATATTCATCTCCTGCAAATTTCCC	TTTAACTGATAAACCGACTGACAG

**TABLE 4:** Genotyping results of candidate SNPs and their *p* values of GLM analyses.

ID of SNPs	Genotypes (number)	Phenotypes (mean ± SD)	Allele (frequency)	*p* value in GLM
SNP3085_190487	TT(186)	8.87 ± 2.84	T(0.75)	7.88E–05*
	CT(81)	9.86 ± 2.98	C(0.25)	
	CC(36)	11.04 ± 3.13		
SNP1678_860109	GG(233)	9.76 ± 3.01	G(0.88)	1.05E–04*
	GA(70)	8.19 ± 2.64	A(0.12)	
	AA(0)	–		
SNP3406_465799	AA(241)	9.65 ± 3.01	A(0.89)	3.28E–03*
	AG(54)	8.15 ± 2.80	G(0.11)	
	GG(6)	10.04 ± 1.70		
SNP2570_399791	GG(213)	9.49 ± 2.88	G(0.84)	5.05E–03*
	GC(73)	8.72 ± 3.12	C(0.16)	
	CC(12)	11.43 ± 3.12		
SNP3424_278362	GG(194)	8.91 ± 2.97	G(0.76)	7.02E–03
	GA(14)	7.84 ± 2.86	A(0.24)	
	AA(55)	10.11 ± 2.62		
SNP555_246199	CC(264)	9.55 ± 3.00	C(0.93)	2.13E–02
	CG(34)	8.57 ± 2.84	G(0.07)	
	GG(4)	6.27 ± 1.12		
SNP629_59929	TT(161)	9.59 ± 3.06	T(0.72)	1.26E–01
	TA(110)	9.36 ± 2.39	A(0.28)	
	AA(31)	8.38 ± 2.88		
SNP2315_402243	GG(123)	9.10 ± 3.09	G(0.62)	3.49E–01
	AG(121)	9.65 ± 2.89	A(0.32)	
	AA(54)	9.40 ± 2.97		

The asterisk (*) indicates a significant correlation with BW after Bonferroni correction (p < 6.25E–03).

### Annotation of Significant SNPs

The SNPs showing significant correlations with BW after Bonferroni correction were further analyzed and annotated. The flanking genes around the significant SNPs are listed in Table 5. Four growth-associated SNPs were found to be located near the genes. There were two unknown genes around the most significant SNP3085_190487, and the second most significant SNP1678_860109 was located in intron 2 of an unknown gene. The other two candidate growth-associated genes near the SNP3406_465799 and SNP2570_399791 were predicted as *deoxycytidylate deaminase* (*dCMPD*) and *non-receptor protein tyrosine kinase* (*NPTK*). Distances from growth-associated SNPs to candidate genes ranged from 2.6 to 10.0k. Particularly, there were seven transcript variants for *L. vannamei NPTK* and two for *dCMPD* in the NCBI GenBank. By comparing sequences of transcripts, we found that the sequence variation among different transcripts existed in 5′ UTR for both genes.

**TABLE 5:** Gene annotations and the distances from significant SNPs to candidate genes (bp). Positive indicates gene locates in downstream of the SNP, and negative indicates in upstream.

ID of significant SNPs	Annotated candidate gene	Distance from significant SNP
SNP3085_190487	Two unknown genes	−2.8 k and +9.8 k, respectively
SNP1678_860109	Unknown gene	Located in intron 2
SNP3406_465799	Deoxycytidylate deaminase (dCMPD)	+2.6 k
SNP2570_399791	Non-receptor protein tyrosine kinase (NPTK)	+10.0 k

### Validation of Growth-Associated SNPs in Candidate Genes by MLM

In this study, the two annotated candidate genes were further analyzed in population 3 to identify the SNPs located in genes. Polymorphisms around 5′ UTR of these two genes were detected by PCR amplification and Sanger sequencing. Two pairs of primers were designed to amplify 5′ UTR for each gene, and the primer sequences are shown in Table 6.

**TABLE 6:** Primer sequences used for identifying polymorphisms in 5′ UTR of *dCMPD* and *NPTK*.

Genes	Fragment length (bp)	Primer sequence (5′–3′)
*dCMPD*	924	1F: CTTCATGCACAGAAGTGAAGGAC 1R: ACAGCTAACTTCCTGTCATGGAA
		2F: GTCTCCTGATGTCCCAATATCGA 2R: CCATTTCCCTCTATCTACGCTCA
*NPTK*	922	1F: ACGTAACTCACTATCGGTTTGGT 1R: GAAGTGATACGGGAAATGCTGAC
		2F: TGTGACGAGTAAAACGCTGAGT 2R: TGGCCTACCAACAAGAACCATA

High polymorphisms were detected in amplified fragments of *dCMPD* and *NPTK.* Roughly, there were more than 45 SNPs and 5 indels in the amplified fragments of *dCMPD* and more than 32 SNPs and 2 indels in those of *NPTK.* However, most SNPs could be genotyped only in a few samples by Sanger sequencing due to the existence of the indels. Finally, 12 SNPs in *NPTK* and 8 SNPs in *dCMPD* with MAF > 0.01 were used to detect their associations with BW. The *p* values for each SNP are shown in Table 7. One SNP within intron 1 of *NPTK* (NPTK-278) and two SNPs located about 100 bp upstream of exon 1 of *dCMPD* (dCMPD-118 and dCMPD-129) were found significantly correlated with BW (*p* < 0.01).

**TABLE 7:** Alleles and *p* values of MLM association analyses between each SNP and BW.

ID of SNPs	Alleles	*p* value	ID of SNPs	Alleles	*p* value
NPTK-211	T > A	0.313	NPTK-274	A > G	0.146
NPTK-217	T > G	0.052	NPTK-278	A > C	0.002*
NPTK-225	C > G	0.428	dCMPD-88	C > T	0.171
NPTK-226	G > T	0.089	dCMPD-89	C > T	0.113
NPTK-228	C > T	0.549	dCMPD-100	C > A	0.121
NPTK-236	C > T	0.705	dCMPD-118	A > G	0.000*
NPTK-237	A > C	0.121	dCMPD-119	C > T	0.198
NPTK-241	G > T	0.567	dCMPD-122	G > T	0.128
NPTK-242	A > G	0.195	dCMPD-125	A > T	0.984
NPTK-245	C > A	0.405	dCMPD-129	A > G	0.008*

The numbers in SNP ID represent locations in amplified fragments. The asterisk (*) indicates a significant correlation with BW (p < 0.01).

Linkage disequilibrium analyses among these significant SNPs showed that dCMPD-118 and dCMPD-129 were in strong LD (Table 8), which indicates an overlap exists between their effects on BW. Therefore, the SNP with a larger effect, dCMPD-118, was included for further analysis. The least squares mean, additive, dominance, and substitution effects of NPTK-278 and dCMPD-118 are shown in Table 9. An overdominance effect was found in *dCMPD.*

**TABLE 8:** Linkage disequilibrium (LD) analysis results among three associated SNPs.

ID of SNPs	NPTK-278	dCMPD-118	dCMPD-129
NPTK-278	–	0	0
dCMPD-118	0.07	–	0.72
dCMPD-129	0.02	0.99	–

*Parameters in the upper triangle were measured with r^2^, and the lower with D′.*

**TABLE 9:** The least squares mean, additive (*a*), dominance (*d*), and substitution (*α*) effects of two independent associated SNPs NPTK-278 and dCMPD-118.

ID of SNPs	Genotypes (number)	Least squares mean	Allele (frequency)	*a*	*d*	*α*
NPTK-278	AA(135)	4.49 ± 2.82	A(0.62)	0.39	0.03	0.39
	AC(118)	4.13 ± 2.22	C(0.38)			
	CC(59)	3.72 ± 1.98				
dCMPD-118	AA(73)	4.42 ± 1.97	A(0.53)	0.27	0.47	0.29
	AG(175)	4.52 ± 2.37	G(0.47)			
	GG(53)	3.79 ± 2.44				

### Comparison Between BLUP and MA-BLUP

Estimated breeding valuess predicted from MA-BLUP and traditional BLUP were compared. For MA-BLUP analysis, we calculated EBVs using three models, two containing the fixed effect of NPTK-278 or dCMPD-118, respectively, and the other one containing both. In particular, the interaction effect between NPTK-278 and dCMPD-118 was not significant, so it was not considered. The correlations among different EBVs are shown in Table 10. The Pearson and Spearman coefficients between different models were high correlated.

**TABLE 10:** Relationship among EBVs from BLUP (model 1) and MA-BLUP (models 2–4). The upper triangle represents Pearson correlation coefficients and the lower triangle represents Spearman correlation coefficients.

	Model 1 (*a+e*)	Model 2 (*u+*s*_1_+e*)	Model 3 (*u+*s*_*2*_+e*)	Model 4 (*u+*s*_1_ + *s*_2_+e*)
Model 1 (*a+e*)	–	0.909	0.878	0.823
Model 2 (*u+*s*_1_+e*)	0.883	–	0.770	0.862
Model 3 (*u+*s*_*2*_+e*)	0.856	0.728	–	0.895
Model 4 (*u+*s*_1_ + *s*_2_+e*)	0.767	0.796	0.844	–

*a is the additive genetic effect; u is the additive genetic effect except for the fixed genotype effects; s_1_ and s_2_ are the fixed genotype effects of NPTK-278 and dCMPD-118, respectively; and e is the random residual.*

The prediction accuracies of all the models including GLMs are listed in Table 11. MA-BLUP containing fixed effects of NPTK-278 and dCMPD-118 (model 4) has the highest accuracy, and those containing one SNP fixed effect (model 2 and model 3) are also larger than the traditional BLUP animal model (model 1). The predicted phenotypes by GLMs (models 5–7) showed weak or no correlation with the observed phenotypes.

**TABLE 11:** Prediction accuracies of BLUP (model 1), MA-BLUP (models 2–4), and GLM (models 5–7).

Model	Prediction accuracy
Model 1 (*a*+*e*)	0.329
Model 2 (*u*+*s*_1_+*e*)	0.342
Model 3 (*u*+*s*_2_+*e*)	0.346
Model 4 (*u*+*s*_1_ + *s*_2_+*e*)	0.360
Model 5 (*s*_1_+*e*)	0.127
Model 6 (*s*_*2*_+*e*)	0.162
Model 7 (*s*_1_ + *s*_2_+*e*)	0.191

*a is the additive genetic effect; u is the additive genetic effect except for fixed genotype effects; s_1_ and s_2_ are the fixed genotype effects of NPTK-278 and dCMPD-118, respectively; and e is the random residual.*

## Discussion

Identification of trait-associated genetic markers or candidate genes is very important both for understanding the molecular mechanisms of phenotypic variation and for breeding applications. In aquaculture species, GWAS analysis was regarded as a useful tool for correlating genetic and phenotypic variations (Abdelrahman et al., 2017). In this study, we present a GWAS analysis to correlate genetic and growth phenotypic variations in *L. vannamei*. Although the results showed that no SNP reached genome-wide significance, a set of SNPs with small *p* values was still worthy of further validation and analysis. This is because the use of more samples and markers would probably increase the confidence levels, theoretically allowing the suggested QTL to be detected at a statistically significant level. Considering that Bonferroni correction was overly restrictive, we selected 50 SNPs with the smallest *p* values as the candidate SNPs for further validation. The association analyses between these SNPs and BW were performed in another population to avoid type I errors. These 50 SNPs were distributed widely in a genome which confirmed that growth trait has a polygenic architecture affected by multiple loci with small effects (Falconer and Mackay, 1996). Further association analysis results proved that some of the most significant SNPs were worthy to be reanalyzed although they did not reach genome-wide significance in GWAS.

After validation of the GWAS result, two candidate growth-associated genes were found, which were *dCMPD* and *NPTK*. dCMPD is an enzyme which catalyzes the deamination of dCMP to dUMP, thus providing nucleotide substrate for thymidylate synthase (Maley and Maley, 1959). Previous studies have indicated its potential importance in DNA replication. Its activity is elevated in such rapidly dividing tissues as embryo (Maley and Maley, 1959), regenerating liver and rat hepatomas (Maley and Maley, 1961). A research on HeLa cell also found that dCMPD showed the highest expression in late S phase and its expression declines in the following G2 phase (Gelbard et al., 1969). There was also a report about the role of *dCMPD* in growth, and inhibition of *dCMPD* could result in inhibition of growth of chick embryo (Roth and Buccino, 1963). Protein tyrosine kinases (PTKs) are enzymes catalyzing the transfer of the gamma-phosphate group of ATP to the hydroxyl groups of specific tyrosine residues in peptides. PTKs are primarily involved in the regulation of cellular functions that are directly related to the multicellular status of the organism, such as growth, differentiation, and cell–cell and cell–extracellular matrix interactions (Robinson et al., 2000). PTKs are divided into two groups according to the presence of transmembrane and extracellular domains, and some lacking these domains are referred to as NPTK (Robinson et al., 2000). So far, there was no report about the function of *dCMPD* and *NPTK* in the growth of *L. vannamei* or other crustaceans.

In *L. vannamei*, there were two transcript variants for *dCMPD* and seven for *NPTK* in NCBI, and sequence variation among different transcripts mainly existed in 5′ UTR for both genes. Besides, considering complex traits are mainly driven by noncoding variants (Pickrell, 2014; Li et al., 2016), polymorphisms around 5′ UTR of these two genes were identified, and their associations with phenotypic variations were detected using MLM analysis. Compared with GLM analysis, the advantage of MLM was accounting for genetic relationships which could avoid confounding effects due to population structure (Kennedy et al., 1992). One SNP in each gene was found significantly correlated with BW. These results showed that *dCMPD* and *NPTK* were growth-associated candidate genes in *L. vannamei* and this deserves further research. However, due to the possible presence of pleiotropy, how allelic frequency changes of these SNPs affect the other important economic traits also needs to be further studied.

Prior to this study, some BW-related genes have been identified in *L. vannamei*, such as *myostatin* (Lee et al., 2015), *protein kinase C delta type*, *ras-related protein Rap-2a* (Yu et al., 2019), *class C scavenger receptor* (Wang et al., 2019), and *MMD2* (Wang et al., 2020). These findings suggested that growth trait has a polygenic architecture and provides the possibility of application of MAS for growth in *L. vannamei*. In this paper, we did not try to uncover the biological functions and mechanisms of the effects on phenotypic variations of these two genes, but to explore the methods on how to apply them in breeding.

For aquaculture species, the primary purpose of GWAS was to identify trait-related genes or markers to apply MAS. However, there has been very few MAS application cases in commercial aquaculture breeding. The biggest obstacle is that for economy traits, almost all of which are complex traits, even the most important loci in the genome have small effects, especially for growth traits. Therefore, the effects of associated genes or markers should be taken as complements to rather than a replacement of polygenic effects (additive genetic effects) in breeding. For the reasons above, MA-BLUP was developed (Fernando and Grossman, 1989) and previous studies showed that MA-BLUP could increase the accuracies of genetic evaluations compared with traditional BLUP (Van Arendonk et al., 1994; Zhang et al., 2003), especially on the condition that marker effect was relatively large (Lande and Thompson, 1990; Villanueva et al., 2005). In this study, there were certain differences between EBVs based on MA-BLUP and BLUP (Table 10). Cross-validation revealed that the performance of MA-BLUP was better than BLUP, and MA-BLUP with two SNP effects was better than with one SNP effect. By using the proposed MA-BLUP method, the prediction accuracy was increased by 9.4%. Besides, the prediction accuracies of GLMs consisting of only fixed effects of SNP were much lower than those of the MA-BLUP or BLUP. This suggested that selection only based on trait-related markers was inefficient.

Since the population for GWAS in this study was relatively small and its genetic background was narrow, the power of GWAS might be weakened. Therefore, we further analyzed the significant SNPs and the candidate genes in the other populations step by step and identified three body weight-related SNPs in two candidate genes. The roles of the two candidate growth genes should be further investigated to enrich our understanding of the genetic basis of growth trait in shrimp. Meanwhile, we give an example of applying the trait-associated SNPs in genetic selection by the MA-BLUP method. The prediction accuracy of MA-BLUP based on significant SNPs was higher than that of traditional BLUP. Foreseeably, as more molecular markers associated with different economic traits were identified, MA-BLUP will have a wide application prospect in the selective breeding of shrimp.

## Data Availability Statement

The datasets presented in this study can be found in online repositories. The names of the repository/repositories and accession number(s) can be found in the article/supplementary material.

## Author Contributions

DL and YY conducted the experiment and data processing. JX and FL conceived and supervised the project. QW and XZ contributed to statistical analysis. ZL and QZ participated in the extraction of genomic DNA. DL, YY, and FL prepared the manuscript. All authors have read and approved the manuscript.

## Conflict of Interest

The authors declare that the research was conducted in the absence of any commercial or financial relationships that could be construed as a potential conflict of interest.

## Footnotes

1 http://www.shrimpbase.net/vannamei.html

2 https://web.expasy.org/translate/
